# Long-term relief of refractory trigeminal neuropathy using high-frequency spinal cord stimulation at the cervicomedullary junction: a 6-year follow-up case report

**DOI:** 10.3389/fnins.2026.1665633

**Published:** 2026-02-20

**Authors:** Daniela Floridia, Rossana Panasiti, Anna Anselmo, Francesco Corallo, Maria Pagano, Irene Cappadona, Salvatore Leonardi, Rocco S. Calabrò

**Affiliations:** IRCCS Centro Neurolesi Bonino-Pulejo, Messina, Italy

**Keywords:** cervicomedullary junction, chronic orofacial pain, high frequency spinal cord stimulation, neuromodulation, trigeminal neuropathy

## Abstract

Chronic neuropathic pain profoundly impairs quality of life and often remains refractory to pharmacological or surgical management. Spinal cord stimulation (SCS) is considered a second-line therapy when conventional treatments fail. In this context, high-frequency spinal cord stimulation (HFSCS) targeting the cervicomedullary junction (CMJ) has emerged as a promising option for drug-refractory facial pain syndromes, including trigeminal neuropathy, though clinical evidence remains limited. We report the case of a 67-year-old woman who developed severe right-sided trigeminal neuropathic pain following petroclival meningioma surgery. After multiple unsuccessful interventions, she underwent implantation of a 10 kHz HFSCS system targeting the CMJ. An epidural lead was placed at the C1-C2 level and connected to an implantable pulse generator, delivering continuous stimulation. The procedure produced complete relief of paroxysmal electric shock-like pain and neurophysiological evidence of reduced trigeminal nociceptive activity. Analgesia was sustained for 6 years, with a transient relapse due to battery depletion, which resolved completely after generator replacement. These findings confirm the long-term efficacy and durability of CMJ-targeted HFSCS and highlight the importance of structured follow-up and device maintenance. HFSCS at the CMJ may represent a safe and durable therapeutic option for refractory trigeminal neuropathy, warranting validation through larger prospective studies.

## Introduction

Chronic pain affects millions of individuals worldwide, substantially impairing quality of life and functional capacity. It may originate from a wide range of pathological conditions, including peripheral nerve disorders such as complex regional pain syndrome and primary pain syndromes, including neuropathic pain and fibromyalgia (Zolezzi et al., 2022). Over recent decades, multiple medical and surgical strategies have been developed to manage chronic pain. Nevertheless, a significant proportion of patients fail to achieve adequate symptom control with first-line therapies. In these cases, advanced neuromodulatory interventions commonly referred to as second-line approaches are required. Among these, spinal cord stimulation (SCS) represents a well-established technique that delivers electrical impulses to specific spinal segments to modulate nociceptive transmission (Gupta et al., 2022). SCS exerts its therapeutic effect through neuromodulation of ascending pain pathways, altering the excitability of afferent neurons and modifying the perception of pain. Depending on the etiology and distribution of pain, stimulation may be applied either at the segmental spinal level or at the cervicomedullary junction (CMJ) (Li et al., 2022). Cervical SCS is generally indicated for conditions such as brachial plexus injury, complex regional pain syndrome, degenerative disc disease, failed neck surgery syndrome, chronic radiculopathy, and post-herpetic neuralgia (PHN). Conversely, CMJ SCS is primarily utilized in refractory craniofacial pain syndromes, including trigeminal deafferentation pain, trigeminal neuropathic pain, and occipital neuralgia (Texakalidis et al., 2019). The neuroanatomical rationale for CMJ SCS lies in its influence on the spinal trigeminal tract and nucleus, particularly the caudal spinal nucleus (SpC), which contains second-order neurons responsible for relaying nociceptive and thermal information from the ipsilateral face. Sensory neurons within the trigeminal ganglion (TG) provide afferent innervation to the face, scalp, and associated sensory structures such as the teeth, sinuses, dura mater, cornea, and temporomandibular joint (TMJ) (Mills et al., 2021; Kim et al., 2024). Nociceptive stimuli arising in the orofacial region are transmitted by TG neurons to the SpC, which also receives convergent afferents from cranial nerves VII, IX, and X. Despite growing interest in neuromodulatory interventions, the role of SCS applied at the CMJ level in managing facial pain remains largely underexplored (Middleton et al., 2021). Notably, Tomycz et al. (2011) reported one of the first successful applications of CMJ stimulation for head and facial pain, demonstrating significant clinical improvement (Tomycz et al., 2011). However, the precise neurophysiological mechanisms underlying this therapeutic approach remain incompletely understood. In this context, we report a case illustrating the successful application of 10 kHz high-frequency spinal cord stimulation (HFSCS) targeting the CMJ in a patient with right-sided cranial trigeminal neuropathy.

## Case report

### Summary of clinical history

**Figure 1:**
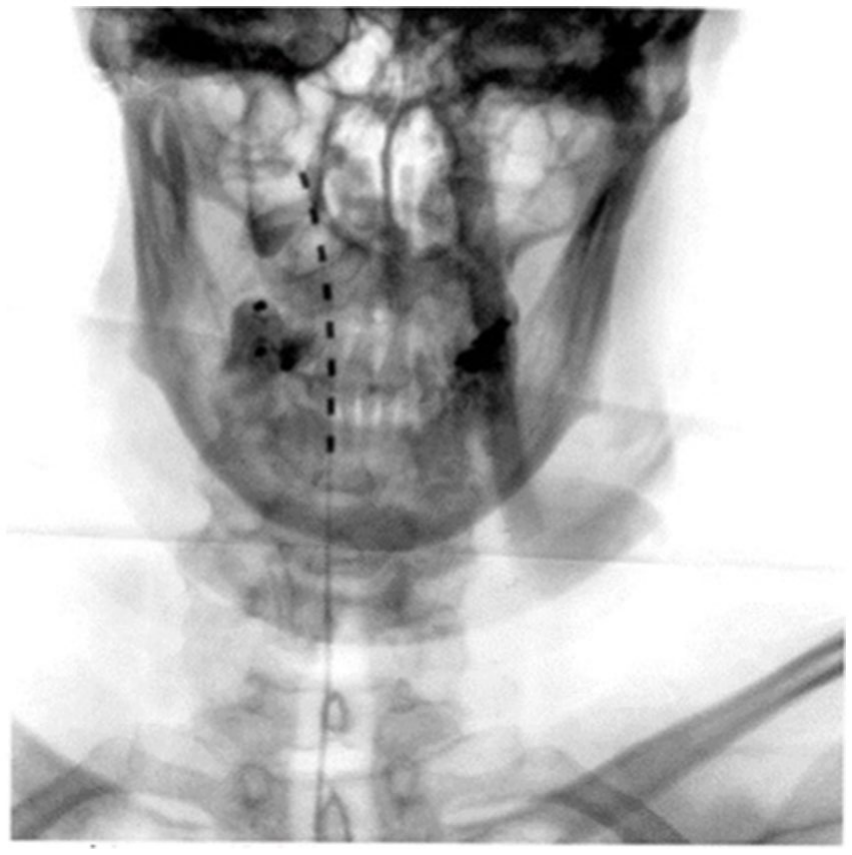
Radiographic image showing the correct placement of the epidural 8-contact lead at the cervicomedullary junction (C1–C2 level) in the anteroposterior view.

In 2004, a 67-year-old woman began experiencing severe, persistent right-sided facial pain characterized by paroxysmal, electric shock-like episodes interspersed with continuous burning and numbness in the maxillary and mandibular divisions of the trigeminal nerve (V2–V3) (Floridia et al., 2020). The pain was triggered by mild tactile stimulation of the cheek and jaw regions and was associated with marked sensory loss and dysesthesia. Initial pharmacological management with carbamazepine and gabapentin failed to provide adequate pain control, and dental interventions, including multiple tooth extractions, were performed under the assumption of odontogenic pain but yielded no benefit. The clinical presentation was consistent with painful trigeminal neuropathy, rather than classical trigeminal neuralgia, given the presence of continuous burning pain and objective sensory deficits. She later experienced symptoms of orthostatic hypotension, episodes of blurred vision and dysphagia. MRI revealed a petroclival meningioma, which was surgically removed in 2006. Following the surgical procedure, the patient developed multiple postoperative complications, including cranial nerve deficits, chronic orofacial pain, dysarthria, and herpes-related sequelae. The persistent orofacial pain led to numerous therapeutic interventions, including thermorhizotomy, intrathecal morphine infusion (later replaced with ziconotide), and radiosurgery. However, these treatments resulted in minimal or no sustained pain relief. In 2013, a cervical spinal cord stimulator (SCS) was implanted in an attempt to achieve better analgesic control. The device consisted of a percutaneous epidural lead positioned at the C2–C4 level, connected to an implantable pulse generator (IPG) placed in the right infraclavicular region. The system used a non-rechargeable battery (Medtronic, Model 37,702). Despite appropriate programming adjustments, including tonic stimulation settings with pulse widths of 300 μs and frequencies between 40 and 60 Hz, the therapy was discontinued due to bothersome paresthesia and the absence of meaningful pain relief. By 2017, the patient continued to suffer from severe, intractable trigeminal pain, leading to substantial functional impairment and significant disruption of sleep quality. In December 2018, a high-frequency spinal cord stimulation (HFSCS) system was implanted at the cervicomedullary junction. The permanent system consisted of an epidural 8-contact lead (Nevro Senza®, Nevro Corp., Redwood City, CA, USA) connected to a rechargeable pulse generator implanted in the right abdominal region. The device was programmed for high-frequency stimulation (10 kHz, pulse width 30 μs, amplitude 1.2–1.8 mA, subperception stimulation). Precise stimulation parameters included intensity titrated to sub-threshold sensory levels (mean ~1.5 mA), pulse width 30 μs, frequency 10 kHz, and bipolar configuration with lead placed at C1–C2 under fluoroscopic control. Shortly after implantation, the patient experienced marked improvement in electric shock-like pain in the right cheek. Complete pain relief was achieved within 15 days, restoring chewing function and allowing oral hygiene without triggering pain. A dull background pain emerged, consistent with chronic central neuropathic pain, successfully managed with pregabalin. Follow-up confirmed clinical improvement including sleep, mood, and psychosocial well- being. Neuropathic pain was evaluated using the nociceptive blink reflex (nBR). Stimulation was delivered over the supraorbital branch (V1) at 1.2 × pain threshold (≈8–12 mA), pulse duration 0.2 ms, frequency 0.2–0.3 Hz, 20 stimuli; EMG recorded from orbicularis oculi with 10–1,000 Hz filter. At baseline, R2 latency 40–46 ms (normal range) and normal waveform morphology were observed, but absence of habituation (0%) indicated trigeminal hyperexcitability. Upon HFSCS activation: R2 amplitude decreased by 27% (from ~320 μV to ~235 μV); Partial recovery of habituation (~45%); Non-nociceptive blink reflex remained intact, confirming preserved sensory function. These findings demonstrate an active modulatory effect on trigeminal nociceptive pathways and central pain processing. Pain remained 0/10, confirming the durable benefit of HFSCS. Radiographic verification confirmed the correct positioning of the epidural 8-contact lead at the cervicomedullary junction (C1–C2 level), as shown in Figure 1.

### Follow-up

During the follow-up period, the patient showed sustained clinical improvement under continuous high-frequency spinal cord stimulation (HFSCS). As summarized in Table 1, complete resolution of paroxysmal electric shock-like facial pain [Numerical Rating Scale (NRS) = 0] was achieved shortly after implantation and maintained at 3-month, 2-year, and 4-year follow-up evaluations. This was associated with marked improvements in daily functioning, sleep quality, and psychosocial well-being. Health-related quality of life progressively improved across multiple SF-36 domains, accompanied by reductions in anxiety and depressive symptoms as measured by HADS and BDI-II scores. Cognitive performance assessed by the MoCA remained overall stable with mild fluctuations over time. At the 6-year follow-up, the patient reported recurrence of severe facial pain characterized by electric shock–like sensations and allodynia, predominantly affecting the V2 and V3 distributions of the trigeminal nerve. Pain intensity returned to 10 on the NRS and was associated with dysphagia, masticatory difficulties, and sleep disruption, closely resembling the pre-implant clinical presentation. Psychometric reassessment demonstrated worsening anxiety and depressive symptoms and a decline in quality-of-life measures. A subsequent technical evaluation revealed complete depletion of the implantable pulse generator battery due to irregular charging, resulting in interruption of stimulation. Following battery recharge and reactivation of stimulation, the patient experienced immediate and complete pain relief (NRS = 0), with rapid improvement in sleep, mood, and overall functioning. At the most recent follow-up visit (June 2024), sustained complete pain relief was confirmed under continuous high-frequency stimulation. Throughout the entire follow-up period, no device-related complications were observed, including lead migration, infection, hardware malfunction, or stimulation- induced paresthesia.

**Table 1:** Longitudinal clinical and psychometric outcomes during follow-up.

Outcome	Baseline	3-month follow- up (post-implant)	2-year follow-up	4-year follow-up	6-year follow-up (battery depletion)	Post-recharge (6-year)
NRS Pain	10	0	0	5	10	0
SF-36 Physical	30	50	52	50	20	55
SF-36 Physical Role	20	60	62	61	16	65
SF-36 General Health	27	64	61	64	24	67
SF-36 Bodily Pain	29	68	67	62	18	69
SF-36 Social Functioning	35	54	56	55	27	58
SF-36 Energy/Vitality	40	50	52	51	30	55
SF-36 Emotional	35	58	60	59	26	62
SF-36 Mental	27	61	63	62	35	65
MoCA	16	20	21	20	15	22
HADS-Anxiety	38	24	23	24	40	22
BDI-II	51	26	25	26	45	20

## Discussion

The extended duration of follow-up represents a major strength of the present case report, as long-term data on neuromodulation for refractory trigeminal neuropathic pain remain scarce in the literature. Chronic neuropathic pain is a dynamic condition, and sustained observation over several years is essential to assess durability of analgesic benefit, stability of functional improvement, and the emergence of delayed adverse events (Isagulyan et al., 2023; Rauck et al., 2023; Caylor et al., 2019). Nevertheless, some methodological limitations must be acknowledged. Follow-up assessments were not conducted at predefined intervals, and standardized outcome measures were applied retrospectively. Consequently, psychometric instruments such as the SF-36, BDI-II, and MoCA should be interpreted with caution, as their longitudinal variability may be influenced by multiple confounding factors, including emotional state, disease chronicity, and long-term pharmacological exposure (Caylor et al., 2019). Despite these limitations, the magnitude and consistency of clinical improvement observed after implantation strongly support the clinical relevance of the intervention. Improvements in health-related quality of life and affective symptoms exceeded published minimal clinically important differences for the SF-36 and BDI-II in several domains, indicating a meaningful benefit beyond statistical fluctuation (Petersen et al., 2023; Patel et al., 2023). In contrast, changes in cognitive performance assessed by the MoCA were more modest and variable over time. This finding is consistent with previous evidence suggesting that cognitive scores in patients with chronic pain may fluctuate as a function of pain burden, mood, sleep quality, and medication effects, rather than reflecting a direct neuromodulatory impact on cognition itself (Mills et al., 2021; Caylor et al., 2019). Taken together, the longitudinal psychometric profile supports a sustained global benefit of high-frequency spinal cord stimulation, while highlighting the inherent complexity of long-term outcome assessment in chronic pain populations (Kapural et al., 2016; Malik et al., 2024). The contribution of concomitant pregabalin therapy to the observed clinical outcomes also warrants careful discussion. Pregabalin is a first-line pharmacological agent for neuropathic pain and has demonstrated efficacy across a range of peripheral and central neuropathic conditions (Joosten and Franken, 2020). In the present case, pregabalin was introduced to manage a residual dull, background pain component that became apparent after resolution of the paroxysmal electric shock–like attacks. This phenomenon is not uncommon in trigeminal neuropathic pain, where distinct pain generators may coexist and become unmasked following suppression of the dominant paroxysmal component. However, several observations support the conclusion that high-frequency spinal cord stimulation represented the primary driver of sustained analgesia (Zuidema et al., 2023). Most notably, recurrence of severe pain occurred exclusively during periods of stimulation interruption caused by complete battery depletion, and pain intensity rapidly returned to baseline levels (NRS = 10) despite continuation of the pharmacological regimen. Restoration of stimulation resulted in immediate and complete pain relief without any adjustment of pregabalin dosage. This tight temporal coupling between stimulation status and pain control provides strong causal evidence for the central role of continuous high-frequency neuromodulation, consistent with previous reports demonstrating the dependence of analgesic efficacy on uninterrupted stimulation in long-term HFSCS therapy (Petersen et al., 2023; Patel et al., 2023; Provenzano et al., 2023). Accordingly, pregabalin should be regarded as an adjunctive component of a multimodal treatment strategy rather than the principal determinant of outcome (Piacentino et al., 2025; Alvarez et al., 2025; González-González et al., 2025). This distinction is clinically relevant, as it underscores the importance of device maintenance, patient education regarding charging habits, and structured long-term follow-up to prevent inadvertent therapy interruption (Sillevis et al., 2025; Jones and Rosenow, 2020; Maric et al., 2025). From a broader perspective, this case supports the concept that high-frequency spinal cord stimulation at the cervicomedullary junction can provide durable and causally robust analgesia in carefully selected patients with refractory trigeminal neuropathic pain, even when conventional tonic SCS has failed.

## Conclusion

This updated case report demonstrates the sustained long-term efficacy of high-frequency spinal cord stimulation (HFSCS) at the cervicomedullary junction (CMJ) for the treatment of neuropathic pain secondary to trigeminal nerve injury that was unresponsive to both tonic SCS and conventional medical management. The findings highlight the potential of CMJ-targeted HFSCS as a durable and safe neuromodulatory option for patients with refractory trigeminal neuropathic pain. Future prospective and randomized controlled studies are warranted to further validate these observations, assess their impact on patient quality of life, and evaluate the cost–benefit balance of this approach from a public health perspective.

## Data Availability

The raw data supporting the conclusions of this article will be made available by the authors, without undue reservation.
