# Correction: Long-term relief of refractory trigeminal neuropathy using high-frequency spinal cord stimulation at the cervicomedullary junction: a 6-year follow-up case report

**DOI:** 10.3389/fnins.2026.1816472

**Published:** 2026-03-10

**Authors:** Daniela Floridia, Rossana Panasiti, Anna Anselmo, Francesco Corallo, Maria Pagano, Irene Cappadona, Salvatore Leonardi, Rocco S. Calabrò

**Affiliations:** IRCCS Centro Neurolesi Bonino-Pulejo, Messina, Italy

**Keywords:** cervicomedullary junction, chronic orofacial pain, high frequency spinal cord stimulation, neuromodulation, trigeminal neuropathy

An incorrect **Funding** statement was provided. The correct funder is Ministry of Health, Italy Current Research Funds 2025 and the correct **Funding** statement reads:

The author(s) declared that financial support was received for this work and/or its publication. This study was supported by Current Research Funds 2025, Ministry of Health, Italy.

The original version of this article has been updated.

